# Supplementary material for: A Site-Ordered Quadruple Perovskites, RMn3Ni2Mn2O12 with R = Bi, Ce, and Ho, with Different Degrees of B Site Ordering
Source: Molecules. 2025 Apr 14;30(8):1749. doi: 10.3390/molecules30081749 (PMC12029892; doi:10.3390/molecules30081749)
Supplement: Supplementary file 1 [file molecules-30-01749-s001.zip › molecules-3580851-supplementary.pdf]

Supporting Information

A-site-ordered Quadruple Perovskites, $RMn_3Ni_2Mn_2O_{12}$ with $_2$R = Bi, Ce, and Ho, with Different Degrees of B-site Ordering

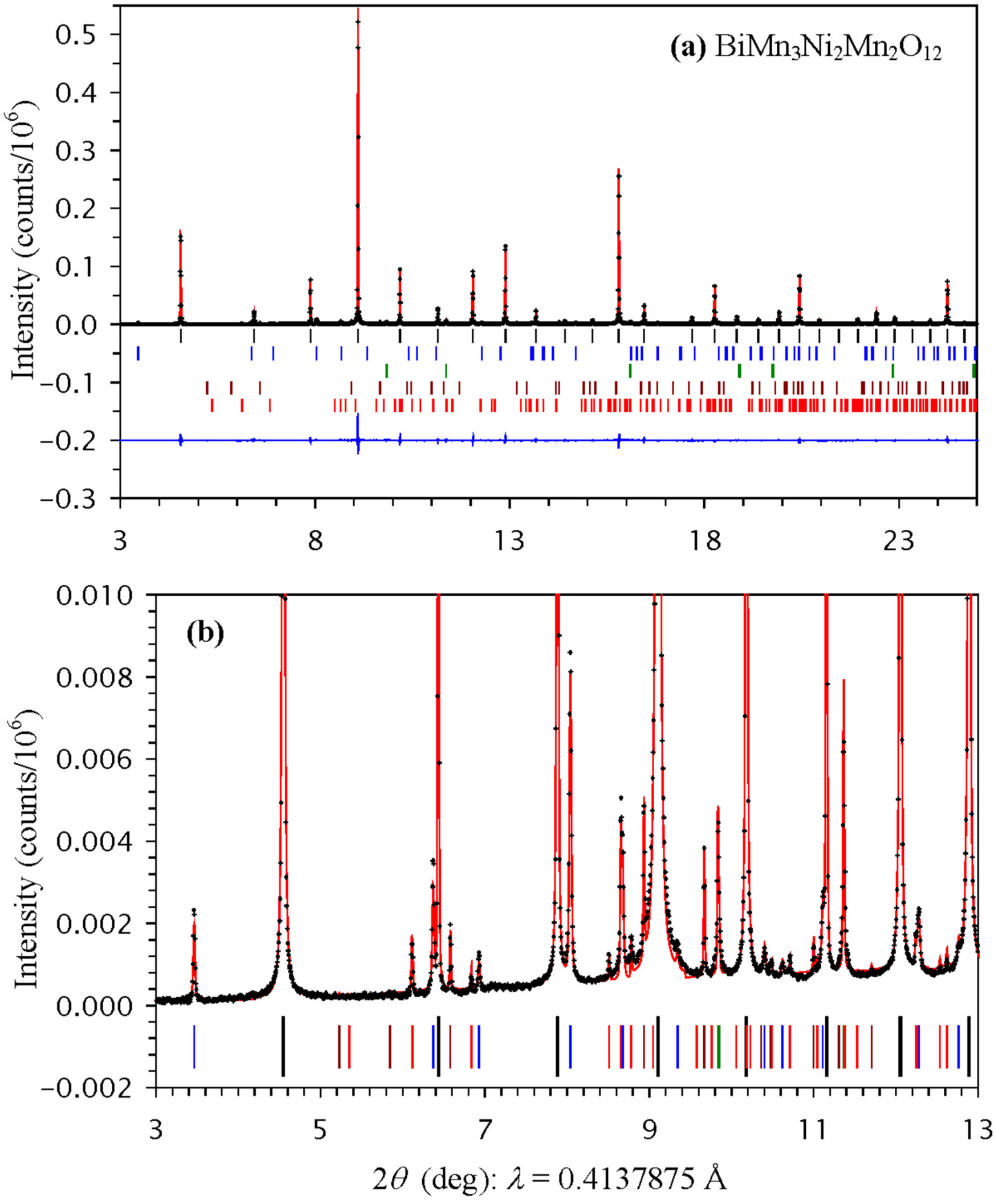

Figure S1. (a) Fragments of experimental (black crosses), calculated (red line), and difference (blue line at the bottom) room-temperature synchrotron X-ray powder diffraction patterns of $BiMn_3Ni_2Mn_2O_{12}$ (the *Im*−3 modification) in a 2θ range of 3° to 25°. The tick marks show possible Bragg reflection positions for the main phase (black) and impurities (from top to bottom for $Bi_2O_2CO_3$ (blue; 1.4 wt. %), NiO (green; 1.9 wt. %), $NiMnO_3$ (brown; 2.0 wt. %), and $GdFeO_3$-type (red; 0.5 wt. %; a = 5.5767 Å, b = 7.7491 Å, and c = 5.4052 Å)). (b) Magnified parts in a 2θ range of 3° to 13°.

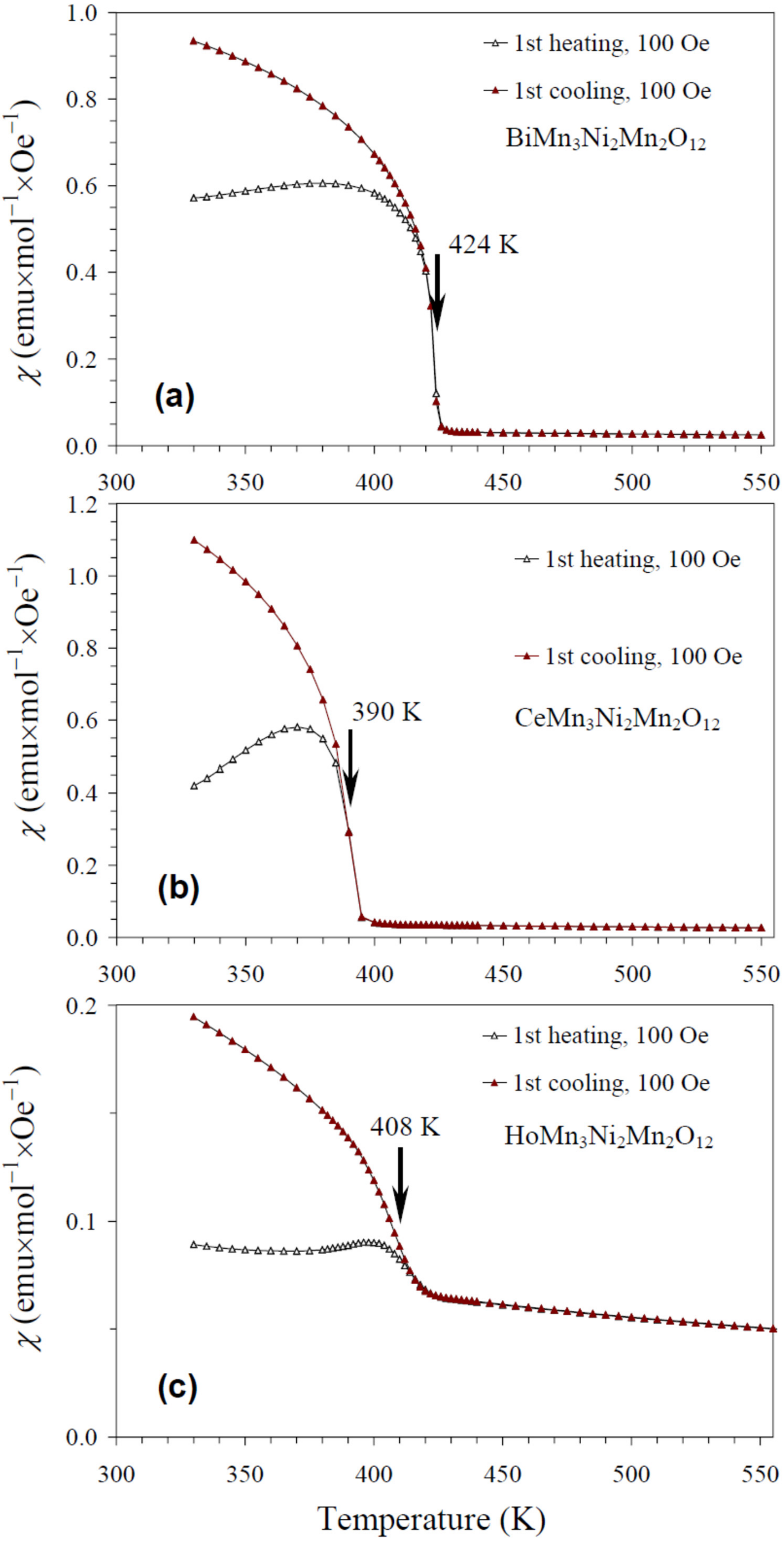
1st heating, 100 Oe
1st cooling, 100 Oe
BiMn3Ni2Mn2O12
424 K
(a)
1st heating, 100 Oe
1st cooling, 100 Oe
CeMn3Ni2Mn2O12
390 K
(b)
1st heating, 100 Oe
1st cooling, 100 Oe
HoMn3Ni2Mn2O12
408 K
(c)
χ (emu×mol⁻¹×Oe⁻¹)
Temperature (K)

Figure S2. Zero-field-cooled (empty symbols; 1st heating) and field-cooled (filled brown symbols; 1st cooling) dc magnetic susceptibility curves ($\chi = M/H$) of (a) $BiMn_3Ni_2Mn_2O_{12}$, (b) $CeMn_3Ni_2Mn_2O_{12}$, and (c) $HoMn_3Ni_2Mn_2O_{12}$, measured at H = 100 Oe between 330 K and 550 K. ⊚ rrows show magnetic transitions in $NiMnO_3$ impurity: different transition temperatures suggest that real chemical compositions of this impurity slightly vary in different samples. The χ values suggest that the amount of $NiMnO_3$ impurity in $HoMn_3Ni_2Mn_2O_{12}$ was about 5 times smaller than in $BiMn_3Ni_2Mn_2O_{12}$ and $CeMn_3Ni_2Mn_2O_{12}$. The 2nd heating-cooling cycle was performed at H = 10 kOe; the 3rd heating-cooling cycle was performed at H = 70 kOe (see the main text).
